# Harm avoidance is associated with progression of parkinsonism in community-dwelling older adults: a prospective cohort study

**DOI:** 10.1186/1471-2318-14-54

**Published:** 2014-04-23

**Authors:** Aron S Buchman, Lei Yu, Robert S Wilson, Joshua M Shulman, Patricia A Boyle, David A Bennett

**Affiliations:** 1 Rush Alzheimer’s Disease Center, Rush University Medical Center, Chicago, IL, USA; 2 Department of Neurological Sciences, Rush University Medical Center, Chicago, IL, USA; 3 Department of Behavioral Science, Rush University Medical Center, Chicago, IL, USA; 4 Departments of Neurology and Molecular and Human Genetics, Baylor College of Medicine, Houston, TX, USA; 5 Jan and Dan Duncan Neurological Research Institute, Houston, TX, USA

**Keywords:** Late-life motor impairment, Aging, Parkinsonism, Harm avoidance

## Abstract

**Background:**

We tested the hypothesis that harm avoidance, a trait associated with behavioral inhibition, is associated with the rate of change in parkinsonism in older adults.

**Methods:**

At baseline harm avoidance was assessed with a standard self-report instrument in 969 older people without dementia participating in the Rush Memory and Aging Project, a longitudinal community-based cohort study. Parkinsonism was assessed annually with a modified version of the motor section of the Unified Parkinson’s Disease Rating Scale (mUPDRS).

**Results:**

Average follow-up was 5 years. A linear mixed-effects model controlling for age, sex and education showed that for an average participant (female, 80 years old at baseline, with 14 years of education and a harm avoidance score of 10), the overall severity of parkinsonism increased by about 0.05 unit/ year (Estimate, 0.054, S.E., 0.007, p <0.001) and that the level of harm avoidance was associated with the progression of parkinsonism (Estimate, 0.004, S.E., 0.001, p <0.001). Thus, for an average participant, every 6 point (~1 SD) increase in harm avoidance score at baseline, the rate of progression of parkinsonism increased about 50% compared to an individual with an average harm avoidance score. This amount of change in parkinsonism over the course of the study was associated with about a 5% increased risk of death. The association between harm avoidance and progression of parkinsonism persisted when controlling for cognitive function, depressive symptoms, loneliness, neuroticism, late-life cognitive, social and physical activities and chronic health conditions.

**Conclusion:**

A higher level of the harm avoidance trait is associated with a more rapid progression of parkinsonism in older adults.

## Background

Late-life parkinsonism including motor slowing (bradykinesia), posture and gait disturbances, rigidity and tremor may be present in up to 50% of community-dwelling older adults without known neurologic disease by age of 85 years. Parkinsonism is associated with a wide range of adverse health outcomes including morbidity, mortality, cognitive decline and dementia [1]. Thus, parkinsonism as part of the spectrum of late-life motor impairment is an important barrier to the maintenance of independence and well-being in old age [2]. Identifying risk factors for the progression of parkinsonism in older adults is an essential step in efforts to develop interventions which decrease its growing burden.

There is increasing recognition that personality traits are important determinants of healthy aging. Harm avoidance is a personality trait indicative of behavioral inhibition [3]. People with a high level of the trait tend to be pessimistic, apprehensive, shy, easily fatigued and risk averse. In prospective studies of children and young adults, low harm avoidance has been associated with worse health-related behavior and health outcomes, possibly because young people with a low level of harm avoidance trait tend to engage in risky behaviors [4,5]. By contrast, in prior works we have shown that in older people, high harm avoidance is associated with incident disability and dementia [6,7]. While there is some evidence to suggest that the level of harm avoidance is related to the level of physical activity, it is not known if or to what extent this trait is associated with other age-related conditions such as parkinsonism [8].

To examine the association of harm avoidance and progression of parkinsonism, we used clinical data from older adults without dementia participating in the Rush Memory and Aging Project [9]. Participants completed a standard self report measure of the trait based on the Harm Avoidance scale from the Temperament and Character Inventory [10]. At baseline and at annual intervals thereafter, they had structured evaluations that included a modified version of the motor section of the Unified Parkinson Disease Rating Scale (mUPDRS) [9]. We tested the hypothesis that a higher level of the harm avoidance trait is associated with the rate of progression of parkinsonism. In further analyses, we examined whether this association was confounded by cognition, other personality traits, psychosocial factors, chronic health conditions and lifestyles.

## Methods

### Participants

Participants were recruited from about 40 retirement facilities and subsidized housing facilities, as well as from church groups and social service agencies in northeastern Illinois. All participants signed an informed consent agreeing to annual clinical evaluation. The study was in accordance with the latest version of the Declaration of Helsinki and was approved by the Rush University Medical Center institutional review board [9].

The Memory and Aging Project began in 1997 and the overall follow-up rate is about 95% of survivors. Because of the rolling admission and mortality, the length of follow-up and number of examinations varies across participants. Further, because the collection of harm avoidance data was not added until 2004, it was only available on a subset of Memory and Aging Project participants. Baseline for these analyses was considered the first cycle at which harm avoidance was assessed. Eligibility for these analyses required: 1) a valid assessment of harm avoidance without evidence of clinical dementia and 2) a valid measure of parkinsonism at the time harm avoidance was assessed and at least one or more follow-up evaluations of parkinsonism in order to assess change in parkinsonism. There were 1,241 participants with harm avoidance assessment and 63 with evidence of clinical dementia were excluded. Of the remaining 1178 participants, there were 209 participants with incomplete parkinsonism data who were excluded from these analyses. These included 19 (1.6%) without any parkinsonism data and 190 (16.1%) who had a single valid assessment of parkinsonism but did not have a second evaluation either because they died before their first follow-up examination or because they had not been in the study long enough for follow-up evaluation leaving 969 participants for these analyses.

### Clinical diagnoses

Clinical diagnoses were made using a multi-step process as previously described [9]. Cognitive function testing included 19 performance tests were summarized into a summary measure of global cognition [9]. Participants were then evaluated in person by an experienced clinician who diagnosed dementia, stroke, or Parkinson’s disease, or other neurological and psychiatric disorders based on published criteria [11-13].

### Assessment of harm avoidance

Study participants completed the 35-items Harm Avoidance scale from the Temperament and Character Inventory assessing harm avoidance, but not the remaining 205 items [3,10]. Items from four subscales were rated as true or false: anticipatory worry (11 items; e.g., “Things often go wrong for me unless I’m careful”; range), fear of uncertainty (7 items; e.g., “I usually feel tense and worried when I have to do something new and unfamiliar”), shyness (8 items; e.g., “I am more shy than most people”), and fatigability (9 items; e.g., “I have less energy and tire more quickly than most people”). The score for the full scale (range, 0–35) and each subscale is the number of item responses indicative of the trait in question. These continuous trait measures were used in analyses as described in a prior study in this cohort [6].

### Assessment of parkinsonism

Trained nurse clinicians administered a 26-item modified motor UPDRS [14]. Four previously established parkinsonian sign scores were derived and scaled from 0 to 100 and a global parkinsonian sign score which was constructed by averaging these four scores was the primary outcome measure in these analyses [14].

### Assessment of other covariates

Age in years was computed from self-reported date of birth, and date of the baseline examination. Sex was recorded at the baseline interview. Education (reported highest grade or years of education) was obtained at baseline testing. Depressive symptoms were assessed with the Center for Epidemiological Studies Depression Scale, using a 10-item (e.g., “I felt sad”) version [15]. Loneliness was assessed with a 5-item (e.g., “I miss having people around”) form of the deJong-Gierveld Loneliness Scale [16]. The neuroticism trait was measured with the standard 48-item (e.g., “I have a low opinion of myself) scale from the NEO Personality Inventory [17]. BMI was calculated based on measured weight and height. Chronic health conditions include 3 vascular risk factors (i.e. hypertension, diabetes mellitus, and smoking), and 4 vascular diseases (i.e., myocardial infarction, congestive heart failure, claudication and stroke) [18]. Frequency of participation in cognitively stimulating activities was quantified with a scale, wherein people rated how often they had participated in each of 7 cognitive activities (e.g., reading a newspaper) over the past year [19]. Frequency of participation in social activity was based on 6 items about activities involving social interaction over the past year [20]. Physical activity was assessed using questions adapted from the 1985 National Health Interview Survey. Minutes spent engaged in each activity were summed and expressed as hours of activity/week [21].

### Statistical analyses

The global parkinsonian sign score had a positively skewed distribution and was subjected to a square root transformation, and the transformed scores were used as outcome variables in all analyses. We first examined pairwise correlations of harm avoidance with several other covariates. Then we used a series of linear mixed-effects models to examine the association of baseline harm avoidance score with the rate of change in severity of parkinsonism during the study period [22]. In these models, repeated measures of square root transformed global parkinsonian sign scores were used as the longitudinal outcome. The primary model predictors included a term for Time in years since the baseline as well as terms for harm avoidance at baseline and a term for its interaction with Time. To control for the effect of demographic variables, we also included terms for age, sex and education and their interaction with Time. The model predicted values, as described in the result section, were derived from the observed values of the independent variables and the corresponding model coefficients and then squared to back-transform these values to the original scale.

In order to contextualize the association of harm avoidance with parkinsonism, we centered baseline age, education, and harm avoidance score, such that all the model coefficients were interpreted with respect to a typical participant, that is, a female 80 years old at baseline, 14 years of education and a harm avoidance score of 10. Specifically, the coefficient for Time was interpreted as the annual rate of change in parkinsonism for a typical participant with the characteristics mentioned above. Similarly, the coefficients for harm avoidance and its interaction with Time estimated the average differences in baseline parkinsonism and rate of change in parkinsonism with a 1-point change in baseline harm avoidance score for a typical participant.

In subsequent models, we examined whether other covariates might account for the association between harm avoidance and parkinsonism. Next, we repeated the global measure of harm avoidance with trait subscores. To determine the clinical significance of the amount of change in the severity of parkinsonism, we first estimated the amount of increase in the rate of progression of parkinsonism with a 1SD increase in harm avoidance score, about 6 points, in a mixed effects model. Subsequently we constructed Cox proportional hazards models examining adverse health consequences of change in parkinsonism and estimated the hazard ratios associated with the amount of increase in rate of progression of parkinsonism, as given above.. These models controlled for age, sex and education. Models were examined graphically and analytically and assumptions were judged to be adequately met. *A priori* level of statistical significance was 0.05. Programming was done in SAS version 9.3 (SAS Institute Inc, Cary, NC) [23].

## Results

### Descriptive properties of harm avoidance measure

The clinical characteristics of the participants included in these analyses at baseline are included in Table 1. Baseline harm avoidance scores ranged from 0 to 34 with higher values indicating higher level of this trait. Harm avoidance scores were approximately normally distributed (mean =10.3; SD = 6.47). Harm avoidance was higher in women (mean = 10.6; SD = 6.56) versus men (mean = 9.1; SD = 6.06) [t[923] = 3.14, p = 0.002].

**Table 1:** Clinical characteristics of the participants included in these analyses at this study’s baseline

**Variable**	**Mean (SD) or N (%)**
Age (years)	80.4(7.42)
Sex (female)	733(75.6%)
Education (years)	14.6(3.09)
BMI*	27.4 (5.37)
Global cognition*	0.2 (0.51)
Depressive symptoms*	1.1 (1.61)
Loneliness*	2.2 (0.59)
Neuroticism*	14.7 (7.08)
**Chronic medical conditions**	
Vascular risk factors*	1.1 (0.81)
Smoking	398 (41.1%)
Diabetes	151 (15.6%)
Hypertension	560 (57.8%)
Vascular diseases*	0.4 (0.71)
Myocardial infarction	109 (11.3%)
Congestive heart failure	56 (6.3%)
Claudication	107 (11.0%)
Stroke	102 (11.6%)
Parkinson’s disease	13 (1.4%)
**Late-life activities**	
Physical activity*	3.3 (3.86)
Cognitive activity*	3.2 (0.67)
Social activity*	2.6 (0.58)

*BMI: Body mass index: weight in kilograms divided by height in meters squared. Global Cognition: composite measure of 19 tests. Depressive Symptoms: Modified 10 item Center for Epidemiological Studies Depression Scale, a higher score indicates greater depressive symptomatology. Loneliness: Loneliness was assessed with a 5-item form of the deJong-Gierveld Loneliness Scale. Neuroticism: was measured with the standard 48-item scale from the NEO Personality Inventory. Vascular Risk Factors: sum of smoking, diabetes, and hypertension self-reported. Vascular Diseases: sum of myocardial infarction, congestive heart failure, claudication and stroke self-reported. Physical Activity: Self-reported frequency of participation in 5 physical activities (hours/week), a higher score indicates more frequent participation. Cognitive Activity: Self reported frequency of participation in 7 cognitive activities, a higher score indicates more frequent participation. Social Activity: Self-reported frequency of participation in 6 items about activities involving social interaction, a higher score indicates more frequent participation.

Harm avoidance was associated with neuroticism (r = 0.50, p < 0.001) depressive symptoms (r = 0.38, p < 0.001), loneliness (r = 0.30, p < 0.001), physical activities (r = -0.13, p < 0.001), cognitive activities (r = -0.16, p < 0.001) and social activities (r = 0.-0.23, p < 0.001), global cognition (r = -0.16, p < 0.001), BMI (r = -0.09, p = 0.005) and vascular diseases (r = 0.06, p < 0.05) but was not associated with vascular risk factors (r = 0.044, p = 0.174).

### Harm avoidance and change in parkinsonism

**Figure 1:**
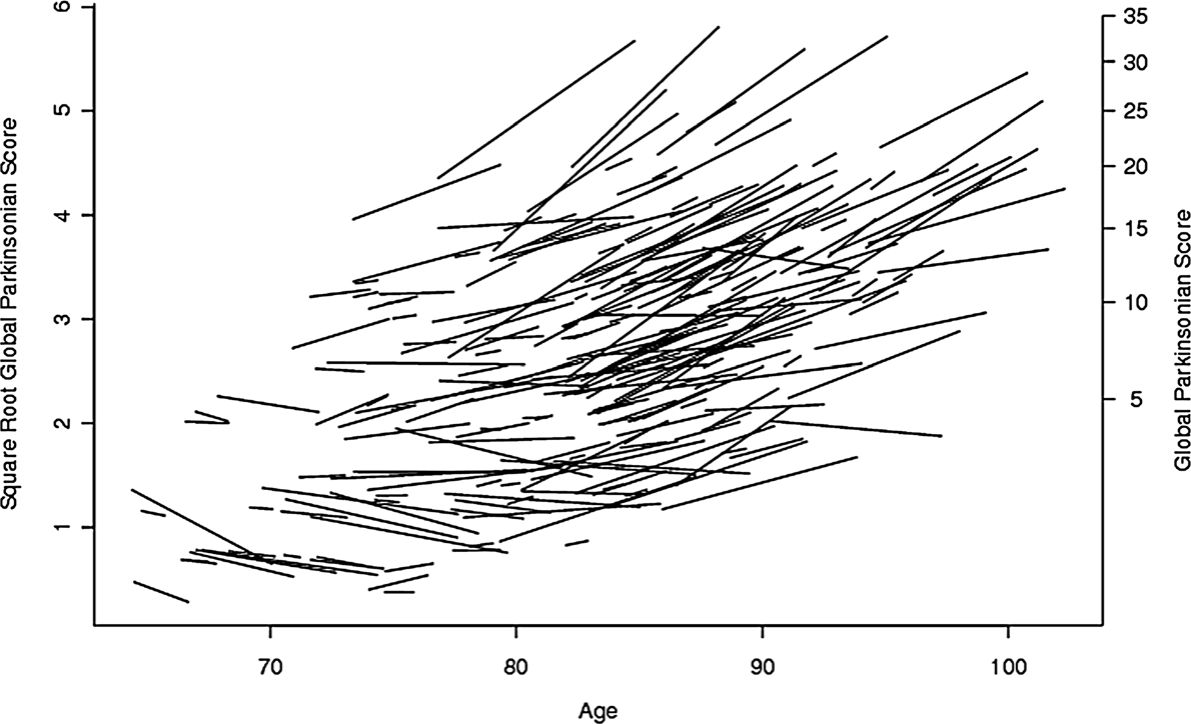
**Person-specific paths of progressive parkinsonism.** The figure is organized according to the age of the participant at each evaluation; the length of each line relative to the x-axis indicates the total years of observation for that individual. The figure is estimated for a 25% random sample of the cohort and shows smoothed person-specific paths estimated from a random-effects model which included a term for time and controlled for age, sex, education and their interaction with time. The left Y axis shows the square root of global parkinsonian scores and the right Y axis shows the untransformed global parkinsonian scores.

Baseline global parkinsonism ranged from 0 to 43 (mean = 7.3; SD = 6.58). We used a linear mixed - effects model controlled for age, sex, and education to test the hypothesis that the baseline level of harm avoidance was associated with the progression of parkinsonism. During an average follow-up of 5 years (mean = 4.8; SD = 2.59 years), for an average participant (female, 80 years old at baseline with 14 years of education and a harm avoidance score of 10) the overall severity of parkinsonism increased by about 0.05 unit/year (Rate of Change, Table 2). Figure 1 illustrates the heterogeneity of the rate of change in parkinsonism for a 25% random sample of the participants included in these analyses. Each line in the figure shows the person-specific change in the rate of parkinsonism during the study. Most participants (59.8%) showed increasing severity of parkinsonism, slope >0, with the remainder exhibiting either less severe parkinsonism, (slope <0, (38.0%) or no change in parkinsonism (2.3%) during the study.

**Table 2:** A model examining the association of baseline harm avoidance with the level and annual rate of change in parkinsonism, adjusting for demographic variables*

**Model term**	**Estimate (S.E., p-Value)**
Annual rate of change in parkinsonism (Time)	0.054 (0.007, <0.001)
Harm avoidance and level of parkinsonism	0.0216 (0.005,<0.001)
Harm avoidance X annual rate of change in parkinsonism	0.004 (0.001, <0.001)
Age and level of parkinsonism	0.068 (0.005, <0.001)
Sex and level of parkinsonism	-0.098 (0.080,0.221)
Education and level of parkinsonism	-0.041 (0.011, <0.001)
Age X annual rate of change in parkinsonism	0.005 (0.001, <0.001)
Sex X annual rate of change in parkinsonism	0.0121 (0.015,0.433)
Education X annual rate of change in parkinsonism	0.005 (0.002,0.024)

*Based on a linear mixed effect model. The model coefficients are interpreted with respect to a female participant 80 years old at baseline, with14 years of education and a harm avoidance score of 10. This model shows the cross sectional association of an average baseline harm avoidance score with parkinsonism at baseline as well as the association of the average harm avoidance score with the annual rate of change in parkinsonism . The model has a total of 9 terms listed in the left column. It contained a term which show the annual rate of change of parkinsonism (Time), the cross-sectional association of in the level of harm avoidance with level of parkinsonism (Harm Avoidance) and its association with the rate of change in parkinsonism (Harm avoidance X Rate of change in parkinsonism). In addition, the model also included 6 additional terms to control for the association of demographic variables (age, sex, education) with level of parkinsonism and their interaction with the annual rate of change in parkinsonism. For each term its Estimate (Standard Error, p Value) is shown in the right column.

Harm avoidance was associated with both the level of baseline parkinsonism (Harm Avoidance, Table 2) and the annual rate of change in parkinsonism (Rate of Change in Parkinsonism, Table 2). Thus, an average participant (female, 80 years old at baseline, with 14 years of education and a harm avoidance score of 10) had an untransformed global parkinsonian score of 6.15 at baseline and an increase of 0.27 units in their parkinsonian score from study entry to their 1 year follow-up assessment. In contrast a similar participant whose baseline harm avoidance score was increased by a 6-point (~1 SD), had an untransformed parkinsonian score of 6.87 at study entry and an increase 0.42 units during their first year in the study, a difference of about 1.6 times larger than the typical participant with the average harm avoidance score.

Since baseline age was also associated with the annual rate of change in parkinsonism in this model (Table 2), we can contextualize the annual rate of change in the severity of parkinsonism associated with harm avoidance by comparing it with the increased severity of parkinsonism associated with a more common metric increased age. Comparing their respective coefficients in this model (Table 2) indicates that a female 80 years old at baseline with14 years of education and a harm avoidance score of 16 (about 1 SD above the mean) had a rate of increasing severity of parkinsonism equivalent to a female 85 years old at baseline, with 14 years of education and a harm avoidance score of 10. [(6 point increase in Harm avoidance score × Estimate for harm avoidance interaction of 0.004)/ Estimate for Age interaction of 0.005) = 4.8 years].

In sensitivity analyses, the association of harm avoidance and the rate of change in parkinsonism remained significant when we excluded individuals with a history of stroke (N = 102, 11.6%) or Parkinson’s disease (N = 13, 1.4%) which might cause more severe parkinsonism (Stroke: Estimate = 0.006, S.E. = 0.001, p < 0.001); PD: Estimate = 0.005, S.E. = 0.001, p < 0.001).

The harm avoidance subscores ranges showed substantial variation: anticipatory worry (range: 0, 11), fear of uncertainty (range: 0,7), shyness (range: 0,8) and fatigability (range: 0,9). To determine whether the subscores showed different associations with the rate of change in parkinsonism, we analyzed each in a separate model. Higher levels of all four subscores were associated with a more rapid progression of parkinsonism (Table 3).

**Table 3:** Association of harm avoidance subscores in a typical participant with the level and the annual rate of change in parkinsonism, adjusted for demographic variables*

**Harm avoidance subscore**	**Annual rate of change in parkinsonism**	**Subscore & level of parkinsonism**	**Subscore X annual rate of change in parkinsonism**
**Anticipatory worry**	0.056 (0.007, <0.001)	0.039 (0.016, 0.017)	0.009 (0.003,0.005)
**Fear of uncertainty**	0.058 (0.008, <0.001)	0.035 (0.021, 0.163)	0.008 (0.004,0.034)
**Shyness**	0.054 (0.008, <0.001)	-0.100 (0.016, 0.51)	0.008 (0.003,0.007)
**Fatigability**	0.061 (0.008, <0.001)	0.119 (0.014, <0.001)	0.010 (0.003,<0.001)

*We repeated the model shown in Table 2, four times replacing harm avoidance with each of its 4 subscores. Each row shows the results for a separate linear mixed effect models for a different subscore. The first term is the annual rate of change in parkinsonism; the 2^nd^ term is the relationship between the subscore and level of parkinsonism and the 3^rd^ columns shows the relationship between the subscore and the annual rate of change in parkinsonism. Each model also included terms (not shown) which controlled for age, sex, education and their interaction with the rate of change in parkinsonism [Estimate (Standard Error, p Value)].

### Potential confounders of harm avoidance and change in parkinsonism

Prior work has linked harm avoidance with cognition, we found that controlling for baseline global cognition, did not affect the association of harm avoidance and progression of parkinsonism (Estimate, 0.004, S.E. 0.001, p < 0.001).

Psychosocial factors can affect late-life motor impairment [16,17]. Therefore we examined whether depressive symptoms, loneliness or the personality trait of neuroticism might affect the association of harm avoidance and increasing parkinsonism. The association of harm avoidance with the rate of change in parkinsonism was not affected when we added terms for depressive symptoms, loneliness or neuroticism (results not shown). With all 3 of these correlated covariates in a single model, the association of harm avoidance and the progression of parkinsonism remained significant (Estimate = 0.003, S.E. = 0.001, p = 0.005).

In further analyses, we considered whether chronic health conditions might have affected our results. Adding terms for BMI and BMI squared (because both very low and very high body mass affect health) as well as for vascular risk factors and vascular diseases, first in separate analyses (results not shown) and then together in a single model did not affect the association of harm avoidance and the progression of parkinsonism (Estimate = 0.005, S.E. = 0.001, p = 0.005).

Since higher levels of late-life activities are associated with a slower rate of motor decline, [20] we examined whether the level of a range of different activities might account for the association of harm avoidance with progression in parkinsonism. In separate analyses (results not shown) as well as together, late-life physical, social and cognitive activities did not affect the association of harm avoidance and progression of parkinsonism (Estimate = 0.003, S.E. = 0.001, p = 0.003).

### Clinical significance of the loss of motor function associated with personality

To determine the clinical significance of the more rapid progression of parkinsonism associated with a 6-point (about 1 SD) increased harm avoidance score at baseline (Harm Avoidance X Time, Table 2), we constructed Cox proportional hazards model examining the association of change in parkinsonism with death and subsequently estimated the hazard ratios associated with the amount of the increased rate of change in parkinsonism attributable to a 6-point increase in baseline harm avoidance score (~1 SD) for an average participant (female 80 years old with 14 years of education). From these models (data not shown), we calculated that a 6-point increased harm avoidance score at baseline for a typical participant was associated with more than a 5% increased risk of death over the course of the study (Hazard Ratio: 1.053, 95% CI: 1.030, 1.077).

## Discussion

Harm avoidance is a broad anxiety-related trait. People with a high level of the trait tend to be pessimistic, apprehensive, shy, and easily fatigued, behaviorally inhibited and to avoid new and potentially aversive situations [3]. In a cohort of more than 900 older adults, those with a high level of the trait showed a more rapid rate of progression of parkinsonism as compared to older adults with a low level of the trait. This association persisted after controlling for other psychosocial factors including depressive symptoms, loneliness and neuroticism, late-life activities including physical, social and cognitive activities, global cognitive function and chronic health conditions. Together these data suggest that the level of the trait of harm avoidance, may identify older individuals at higher risk for more rapidly progressive parkinsonism and provides evidence for the importance of personality traits as one of the growing number of factors which may contribute to healthy aging.

Prior studies have reported that higher levels of harm avoidance are associated with adverse health consequences in older adults including: incident disability, [6] and late-life cognitive impairments including incident mild cognitive impairment (MCI) and Alzheimer’s disease (AD) as well as cognitive decline [7,24,25]. This study extends these findings by showing that harm avoidance is also associated with both the level and rate of progression of parkinsonism in older adults without overt neurologic diseases including dementia, stroke and Parkinson’s disease (PD). Analyses of the sub-scale components of harm avoidance showed that all of its constituent factors are associated with the rate of progressive parkinsonism. The association of harm avoidance and parkinsonism, was robust and remained significant after accounting for related personality traits and psychosocial factors, global cognition, a wide range of late-life activities and chronic health conditions. The results of the current study have important translational implications as they suggest that personality traits need to be considered for our understanding of individual differences in the progression of parkinsonism in older adults. Furthermore, understanding the biologic basis of the association has the potential to lead to new therapeutic targets to reduce the burden of late-life motor impairment.

The basis of the association between harm avoidance and parkinsonism is likely to be complex. While, harm avoidance is related to other personality traits and psychosocial factors, [26] the association of harm avoidance and parkinsonism was unchanged when we adjusted for several of these factors in the current study (Table 3) [27]. Personality traits may affect for lifestyle choices i.e. physical and other late-life activities and could thus link harm avoidance with parkinsonism. In contrast to other personality traits, [17,28] statistical adjustment for late-life physical, cognitive and social activities in the current study did not affect the association of harm avoidance with progressive parkinsonism. Both harm avoidance and motor function in older people are preferentially associated with structural changes in specific brain regions including cortical and cerebellar structures which might account for their association in this study [29-33]. Thus, higher levels of harm avoidance may be lead to stress-related changes in neurotransmitters (e.g., cortisol or dopamine) causing brain atrophy, damaging motor-related brain regions or decreasing the brain’s capacity (motor reserve) to tolerate ongoing neurodegeneration and the accumulation of other neuropathologies [34-37].

Over the years there have been many reports suggesting that PD may be associated with a distinctive personality which may manifest many years before clinical evidence of parkinsonism, but empirical studies have failed to support this suggestion [38,39]. Nonethelss, building on imaging data linking harm avoidance with brain dopamine receptors, recent studies have reported higher levels of harm avoidance occur in individuals with early clinical manifestations of PD such as REM sleep disorder which can manifest years before a clinical diagnosis of PD [40-43]. Studies in the current cohort have suggested that PD pathology is found in up to 40% of older adults without a clinical diagnoses of PD, and these lesions are associated with the severity of parkinsonism proximate to death [14]. Further work is needed to determine if the accumulation of subclinical PD pathology might contribute to the association between harm avoidance and the rate of change in the severity of parkinsonism. Alternatively, lower harm avoidance is associated with resilience, optimism, composure, and energy which may facilitate adaptation to accumulating neurodegeneration and the accumulation of neuropathology in older adults [10,44]. Further work to understand the neurobiologic basis for the current findings has the potential to identify new targets and pathways, for interventions to decrease the growing burden of progressive parkinsonism in older adults.

Our study has some limitations. While some studies have suggested that personality traits such as harm avoidance are stable even in old age, others suggest that personality changes may occur with age but there are few studies which have focused on individuals older than 80 years [45-48]. Further, it is possible that a third unmeasured variable is related to both harm avoidance and progressive parkinsonism. A precondition for participation in the current study was consent to annual exam and organ donation at death, so given the selected nature of the cohort, our findings will need replication in other cohorts. This study was large since on an individual level the effect sizes for the association of harm avoidance and progressive parkinsonism are small. Similar effect sizes have been reported in prior reports of other psychosocial factors with motor decline [17]. Nonetheless, from a public policy perspective even the modest effect sizes observed in the current study are likely to be important.

However, several factors increase confidence in our findings. Perhaps most importantly, the study enjoys high follow-up participation reducing bias due to attrition. In addition, personality traits were assessed among people without dementia and parkinsonism was evaluated as part of a uniform clinical evaluation which incorporated other widely accepted personality, affect and cognitive measures. In addition, a relatively large number of older people were studied, so that there was adequate statistical power to identify the association of interest while controlling for several potentially confounding variables. Results were similar with total score and subscores of the trait of harm avoidance.

## Conclusions

The growing personal and social burden of late-life motor impairment in our aging population is a public health challenge. In a cohort of nearly 1000 older adults, individuals with a higher level of the trait of harm avoidance showed a more rapid rate of progressive parkinsonism as compared to older adults with a low level of the trait. The results of the current study suggest that the level of the trait of harm avoidance may identify older adults at higher risk for more rapid motor decline and underscore that personality traits need to be considered in studies of individual differences of late-life motor impairments. Finally, this study provides additional evidence for the importance of personality traits as one of the growing number of factors which may contribute to healthy aging.

## Competing interests

The authors declare that they have no competing interests.

## Authors’ contributions

ASB, RSW, LY, JMS, PAB, and DAB were involved in the conception, organization and execution of the project. ASB, LY and RSW were involved in the design, execution and review of the statistical analyses. ASB wrote the first draft and RSW, LY, JMS, PAB, and DAB reviewed and critiqued this and subsequent drafts of the manuscript. All authors read and approved the final manuscript. ASB and co-authors had full access to the data, have the right to publish all the data, and have had the right to obtain independent statistical analyses of the data. ASB takes responsibility for the integrity of the data and the accuracy of the data analysis.

## Pre-publication history

The pre-publication history for this paper can be accessed here:

http://www.biomedcentral.com/1471-2318/14/54/prepub

## References

[B1] Louis ED Bennett DA Mild Parkinsonian signs: an overview of an emerging concept Mov Disord 2007 14 12 1681 1688 10.1002/mds.21433 17534951

[B2] Buchman AS Leurgans SE Boyle PA Schneider JA Arnold SE Bennett DA Combinations of motor measures more strongly predict adverse health outcomes in old age: the rush memory and aging project, a community-based cohort study BMC Med 2011 14 42 10.1186/1741-7015-9-42 21507235 PMC3100235

[B3] Cloninger C A systematic method for clinical description and classification of personality variants: a proposal Arch Gen Psychiatry 1987 14 6 573 588 10.1001/archpsyc.1987.01800180093014 3579504

[B4] Hintsanen M Pulkki-Råback L Juonala M Viikari JSA Raitakari OT Keltikangas-Järvinen L Cloninger's temperament traits and preclinical atherosclerosis: the cardiovascular risk in young Finns study J Psychosom Res 2009 14 1 77 84 10.1016/j.jpsychores.2009.01.002 19539821

[B5] Masse Lc TRE Behavior of boys in kindergarten and the onset of substance use during adolescence Arch Gen Psychiatry 1997 14 1 62 68 10.1001/archpsyc.1997.01830130068014 9006402

[B6] Krueger KR Wilson RS Shah RC Tang Y Bennett DA Personality and incident disability in older persons Age Ageing 2006 14 4 428 433 10.1093/ageing/afl028 16788082

[B7] Wilson RS Schneider JA Boyle PA Arnold SE Tang Y Bennett DA Chronic distress and incidence of mild cognitive impairment Neurology 2007 14 24 2085 2092 10.1212/01.wnl.0000264930.97061.82 17562829

[B8] Volkers AC Tulen JHM Duivenvoorden HJ Gieteling MJ Wegewijs-De Jong M Van Den Broek WW Passchier J Pepplinkhuizen L Effect of personality dimensions on the diurnal pattern of motor activity J Pers 2002 14 2 233 248 10.1111/1467-6494.05004 11911121

[B9] Bennett DA Schneider JA Buchman AS Barnes LL Boyle PA Wilson RS Overview and findings from the rush memory and aging project Curr Alzheimer Res 2012 14 646 663 10.2174/156720512801322663 22471867 PMC3439198

[B10] Cloninger CR Przybeck TR Svrakic DM Wetzel RD The Temperament and Character Inventory (TCI): A guide to its development and use 1994 St. Louis, MO: Center for Psychobiology of Personality

[B11] Adams HP Jr Bendixen BH Kappelle LJ Biller J Love BB Gordon DL Marsh EE 3rd Classification of subtype of acute ischemic stroke. Definitions for use in a multicenter clinical trial. TOAST. Trial of Org 10172 in Acute Stroke Treatment Stroke 1993 14 1 35 41 10.1161/01.STR.24.1.35 7678184

[B12] Langston JW Widner H Goetz CG Brooks D Fahn S Freeman T Watts R Core assessment program for intracerebral transplantations (CAPIT) Mov Disord 1992 14 1 2 13 10.1002/mds.870070103 1557062

[B13] Jack CR Jr Albert M Knopman DS McKhann GM Sperling RA Carrillo MC Thies B Phelps CH Introduction to revised criteria for the diagnosis of Alzheimer's disease: National Institute on Aging and the Alzheimer's Association workgroup Alzheimers Dement 2011 14 3 257 262 10.1016/j.jalz.2011.03.004 21514247 PMC3096735

[B14] Buchman AS Shulman JM Nag S Leurgans SE Arnold SE Morris MC Schneider JA Bennett DA Nigral pathology and parkinsonian signs in elders without Parkinson disease Ann Neurol 2012 14 2 258 266 10.1002/ana.22588 22367997 PMC3367476

[B15] Kohout FJ Berkman LF Evans DA Cornoni-Huntley J Two shorter forms of the CES-D (Center for Epidemiological Studies Depression) depression symptoms index J Aging Health 1993 14 2 179 193 10.1177/089826439300500202 10125443

[B16] Buchman A Boyle P Wilson R James B Leurgans S Arnold S Bennett D Loneliness and the rate of motor decline in old age: the rush memory and aging project, a community-based cohort study BMC Geriatr 2010 14 1 77 10.1186/1471-2318-10-77 20969786 PMC2975650

[B17] Buchman AS Boyle PA Wilson RS Leurgans SE Arnold SE Bennett DA Neuroticism, extraversion, and motor function in community-dwelling older persons Am J Geriatr Psychiatry 2013 14 2 145 154 10.1016/j.jagp.2012.10.015 23343488 PMC3406259

[B18] Boyle PA Wilson RS Aggarwal NT Arvanitakis Z Kelly J Bienias JL Bennett DA Parkinsonian signs in subjects with mild cognitive impairment Neurology 2005 14 12 1901 1906 10.1212/01.wnl.0000188878.81385.73 16380610

[B19] Wilson RS Barnes LL Krueger KR Hoganson G Bienias JL Bennett DA Early and late life cognitive activity and cognitive systems in old age J Int Neuropsychol Soc 2005 14 4 400 407 16209420

[B20] Buchman AS Boyle PA Wilson RS Fleischman DA Leurgans S Bennett DA Association between late-life social activity and motor decline in older adults Arch Intern Med 2009 14 12 1139 1146 10.1001/archinternmed.2009.135 19546415 PMC2775502

[B21] Buchman AS Boyle PA Wilson RS Bienias JL Bennett DA Physical activity and motor decline in older persons Muscle Nerve 2007 14 354 362 10.1002/mus.20702 17143881

[B22] Zeger SL Liang KY Albert PS Models for longitudinal data: a generalized estimating equation approach Biometrics 1988 14 4 1049 1060 10.2307/2531734 3233245

[B23] SAS Institute Inc SAS/STAT® Software for Unix, Version (9.18) 2002–2003 Cary, NC: SAS Institute Inc

[B24] Wilson RS Evans DA Bienias JL de Mendes Leon CF Schneider JA Bennett DA Proneness to psychological distress is associated with risk of Alzheimer's disease Neurology 2003 14 11 1479 1485 10.1212/01.WNL.0000096167.56734.59 14663028

[B25] Wilson RS Arnold SE Schneider JA Kelly JF Tang Y Bennett DA Chronic psychological distress and risk of Alzheimer's disease in old age Neuroepidemiology 2006 14 3 143 153 10.1159/000095761 16974109

[B26] Miettunen J Raevuori A A meta-analysis of temperament in axis I psychiatric disorders Compr Psychiatry 2012 14 2 152 166 10.1016/j.comppsych.2011.03.008 21565334

[B27] Wilson RS Boyle PA Buchman AS Yu L Arnold SE Bennett DA Harm avoidance and risk of Alzheimer's disease Psychosomatic Med 2011 14 690 696 10.1097/PSY.0b013e3182302ale PMC3304581 21949425

[B28] Oerlemans WGM Bakker AB Veenhoven R Finding the key to happy aging: a day reconstruction study of happiness J Gerontol B-Psychol 2011 14 6 665 674 10.1093/geronb/gbr040 21724970

[B29] Wright CI Feczko E Dickerson B Williams D Neuroanatomical correlates of personality in the elderly NeuroImage 2007 14 1 263 272 10.1016/j.neuroimage.2006.11.039 17229578 PMC1868480

[B30] Bugg JM Head D Exercise moderates age-related atrophy of the medial temporal lobe Neurobiol Aging 2009 14 506 514 19386382 10.1016/j.neurobiolaging.2009.03.008 PMC2891908

[B31] Jackson J Balota DA Head D Exploring the relationship between personality and regional brain volume in healthy aging Neurobiol Aging 2011 14 2162 2171 10.1016/j.neurobiolaging.2009.12.009 20036035 PMC2891197

[B32] Laricchiuta D Petrosini L Piras F Macci E Cutuli D Chiapponi C Cerasa A Picerni E Caltagirone C Girardi P Tamorri SM Spalletta G Linking novelty seeking and harm avoidance personality traits to cerebellar volumes Hum Brain Mapp 2014 14 1 285 296 10.1002/hbm.22174 22965823 PMC6869029

[B33] Westlye LT Bjornebekk A Grydeland H Fjell AM Walhovd KB Linking an anxiety-related personality trait to brain white matter microstructure: diffusion tensor imaging and harm avoidance Arch Gen Psychiatry 2011 14 4 369 377 10.1001/archgenpsychiatry.2011.24 21464361

[B34] Tuominen L Salo J Hirvonen J Någren K Laine P Melartin T Isometsä E Viikari J Raitakari O Keltikangas-Järvinen L Hietala J Temperament trait harm avoidance associates with μ-opioid receptor availability in frontal cortex: a PET study using [11C]carfentanil NeuroImage 2012 14 3 670 676 10.1016/j.neuroimage.2012.03.063 22484309

[B35] Shibuya N Suzuki A Sadahiro R Kamata M Matsumoto Y Goto K Hozumi Y Otani K Association study between a functional polymorphism of FK506-binding protein 51 (FKBP5) gene and personality traits in healthy subjects Neurosci Lett 2010 14 3 194 197 10.1016/j.neulet.2010.09.010 20849924

[B36] Pallanti S Borgheresi A Pampaloni I Giovannelli F Bernardi S Cantisani A Zaccara G Cincotta M Motor cortex excitability correlates with novelty seeking in social anxiety: a transcranial magnetic stimulation investigation J Neurol 2010 14 8 1362 1368 10.1007/s00415-010-5533-4 20352252

[B37] Waider J Araragi N Gutknecht L Lesch K-P Tryptophan hydroxylase-2 (TPH2) in disorders of cognitive control and emotion regulation: a perspective Psychoneuroendocrinology 2011 14 3 393 405 10.1016/j.psyneuen.2010.12.012 21257271

[B38] Pluck G Brown RG Cognitive and affective correlates of temperament in Parkinson's disease Depress Res Treat 2011 14 8 10.1155/2011/893873 PMC3159022 21869930

[B39] Ishihara L Brayne C What is the evidence for a premorbid parkinsonian personality: a systematic review Mov Disord 2006 14 8 1066 1072 10.1002/mds.20980 16755553

[B40] Poletti M Bonuccelli U Personality traits in patients with Parkinson’s disease: assessment and clinical implications J Neurol 2012 14 6 1029 1038 10.1007/s00415-011-6302-8 22083431

[B41] Postuma RB Gagnon JF Vendette M Montplaisir JY Markers of neurodegeneration in idiopathic rapid eye movement sleep behaviour disorder and Parkinson's disease Brain 2009 14 12 3298 3307 10.1093/brain/awp244 19843648

[B42] Suhara T Yasuno F Sudo Y Yamamoto M Inoue M Okubo Y Suzuki K Dopamine D2 receptors in the insular cortex and the personality trait of novelty seeking NeuroImage 2001 14 5 891 895 10.1006/nimg.2001.0761 11304084

[B43] Yasuno F Suhara T Sudo Y Yamamoto M Inoue M Okubo Y Suzuki K Relation among dopamine D2 receptor binding, obesity and personality in normal human subjects Neurosci Lett 2001 14 1 59 61 10.1016/S0304-3940(01)01552-X 11172939

[B44] Simeon D Yehuda R Cunill R Knutelska M Putnam FW Smith LM Factors associated with resilience in healthy adults Psychoneuroendocrinology 2007 14 8–10 1149 1152 17913377 10.1016/j.psyneuen.2007.08.005

[B45] Small BJ Hertzog C Hultsch DF Dixon RA Stability and change in adult personality over 6 years: findings from the victoria longitudinal study J Gerontol B-Psychol 2003 14 3 P166 P176 10.1093/geronb/58.3.P166 12730309

[B46] Steunenberg B Twisk JWR Beekman ATF Deeg DJH Kerkhof AJFM Stability and change of neuroticism in aging J Gerontol B-Psychol 2005 14 1 P27 P33 10.1093/geronb/60.1.P27 15643035

[B47] Terracciano A Costa PT McCrae RR Personality plasticity after age 30 Personal Soc Psychol Bull 2006 14 8 999 1009 10.1177/0146167206288599 PMC2680603 16861305

[B48] Terracciano A McCrae RR Costa PT Longitudinal trajectories in Guilford Zimmerman temperament survey data: results from the Baltimore longitudinal study of aging J Gerontol B-Psychol 2006 14 2 P108 P116 10.1093/geronb/61.2.P108 PMC2754731 16497954

